# Full Transcriptomic Response of *Pseudomonas aeruginosa* to an Inulin-Derived Fructooligosaccharide

**DOI:** 10.3389/fmicb.2020.00202

**Published:** 2020-02-20

**Authors:** José Manuel Rubio-Gómez, Carlos Molina Santiago, Zulema Udaondo, Mireia Tena Garitaonaindia, Tino Krell, Juan-Luis Ramos, Abdelali Daddaoua

**Affiliations:** ^1^Centro de Investigación Biomédica en Red de Enfermedades Hepáticas y Digestivas, Department of Pharmacology, School of Pharmacy, University of Granada, Granada, Spain; ^2^Department of Microbiology, Instituto de Hortofruticultura Subtropical y Mediterránea “La Mayora”, University of Málaga, Málaga, Spain; ^3^Department of Biomedical Informatics, University of Arkansas for Medical Sciences, Little Rock, AR, United States; ^4^Department of Biochemistry and Molecular Biology II, School of Pharmacy, University of Granada, Granada, Spain; ^5^Department of Environmental Protection, Estación Experimental del Zaidín, Consejo Superior de Investigaciones Científicas, Granada, Spain

**Keywords:** RNA sequencing, rt-qPCR, adhesion, developmental process, molecular transducer, pathogenicity

## Abstract

*Pseudomonas aeruginosa* is an ubiquitous gram-negative opportunistic human pathogen which is not considered part of the human commensal gut microbiota. However, depletion of the intestinal microbiota (Dysbiosis) following antibiotic treatment facilitates the colonization of the intestinal tract by Multidrug-Resistant *P. aeruginosa*. One possible strategy is based on the use of functional foods with prebiotic activity. The bifidogenic effect of the prebiotic inulin and its hydrolyzed form (fructooligosaccharide: FOS) is well established since they promote the growth of specific beneficial (probiotic) gut bacteria such as bifidobacteria. Previous studies of the opportunistic nosocomial pathogen *Pseudomonas aeruginosa* PAO1 have shown that inulin and to a greater extent FOS reduce growth and biofilm formation, which was found to be due to a decrease in motility and exotoxin secretion. However, the transcriptional basis for these phenotypic alterations remains unclear. To address this question we conducted RNA-sequence analysis. Changes in the transcript level induced by inulin and FOS were similar, but a set of transcript levels were increased in response to inulin and reduced in the presence of FOS. In the presence of inulin or FOS, 260 and 217 transcript levels, respectively, were altered compared to the control to which no polysaccharide was added. Importantly, changes in transcript levels of 57 and 83 genes were found to be specific for either inulin or FOS, respectively, indicating that both compounds trigger different changes. Gene pathway analyses of differentially expressed genes (DEG) revealed a specific FOS-mediated reduction in transcript levels of genes that participate in several canonical pathways involved in metabolism and growth, motility, biofilm formation, β-lactamase resistance, and in the modulation of type III and VI secretion systems; results that have been partially verified by real time quantitative PCR measurements. Moreover, we have identified a genomic island formed by a cluster of 15 genes, encoding uncharacterized proteins, which were repressed in the presence of FOS. The analysis of isogenic mutants has shown that genes of this genomic island encode proteins involved in growth, biofilm formation and motility. These results indicate that FOS selectively modulates bacterial pathogenicity by interfering with different signaling pathways.

## Introduction

The human pathogen *Pseudomonas aeruginosa* causes a wide array of life-threatening acute and chronic infections, particularly in immunocompromised, cancer, burn wound, and cystic fibrosis patients (Juhas, 2015). This bacterium is moreover one of the leading causes of nosocomial infections affecting hospitalized patients (Buhl et al., 2015) and mortality associated with hospital-acquired *P. aeruginosa* infectious like ventilator-associated pneumonia or bacteremia is above 35% (Lynch et al., 2017).

Moreover, under continuous antibiotic treatment the intestinal microbiota integrity is compromised and bears depletion of the intestinal microbiota (Dysbiosis), hence, physiological colonization resistance subsequently facilitates the establishment of the *Pseudomonas aeruginosa* in the intestinal ecosystem which might be considered an important internal source for *P. aeruginosa* infection (Ohara and Itoh, 2003; Von Klitzing et al., 2017). It is important to note that pathological alterations of the intestinal microbiota (dysbiosis) is related with continuous antibiotic treatment, obesity, diabetes and fatty liver, and of course alterations of the intestinal barrier function (IBF) as in inflammatory bowel disease and metabolic syndrome (Cano et al., 2013; Miura and Ohnishi, 2014).

The severity and permanence of these infections are related to the ability of *P. aeruginosa* to resist the effect of antibiotics through the formation of biofilms (Mah et al., 2003; Hoiby et al., 2010; Taylor et al., 2014). Important research efforts have been made to study the molecular mechanisms related to the formation maturation and subsequent dispersion of the biofilm (Stoodley et al., 2002; Flemming et al., 2007). A number of surface proteins and appendages, including flagella and type IV pili, were found to be associated with biofilm formation (Klausen et al., 2003; Anyan et al., 2014). Furthermore, this species is characterized by its ability to synthesize the virulent factors exotoxin A and pyocyanin (Ortiz-Castro et al., 2014) that block protein synthesis consequently leading to cell death (Gaines et al., 2007).

Treatment of *P. aeruginosa* infections can be particularly challenging because this bacterium is intrinsically resistant to multiple antibiotics and can easily acquire new resistances (Breidenstein et al., 2011). In fact, over the past three decades, antibiotic resistance among *P. aeruginosa* has escalated globally, *via* the global dissemination of several multidrug-resistant epidemic clones (Miyoshi-Akiyama et al., 2017). *Pseudomonas aeruginosa* infections thus represent a severe threat to human health worldwide and the World Health Organization has declared this bacterium the second priority pathogen for research and development of new strategies to fight it (WHO, 2017).

Besides conventional treatments, one possible strategy is based on the use of functional foods with prebiotic activity which are non-digestible foods (mostly oligosaccharides) that selectively stimulate the growth of a limited number of host-friendly colonic bacteria (Froebel et al., 2019). Thus, from a chemical standpoint, resistance to human digestive enzymes and low absorption are key for these compounds to reach the distal parts of the gut, where they can be fermented by the microbiota, which in turn is selectively modified in the process. These additional actions of prebiotics tend to enhance the capacity of the mucosa to contain luminal microorganisms and their components, i.e., intestinal barrier function (IBF). Normally, passage of microorganisms and/or their components such as Lipopolysaccharides (LPS) to the mucosa and from there to the bloodstream (translocation) is minimal, and the immune system develops tolerance to the microbiota, without inflammation. Conversely, when IBF is compromised translocation ensues, depending on the nature of the dysfunction and the physiological/pathological context. Therefore, inflammation of the intestine is considered to stem from augmented translocation, which engages the adaptive immune system, ultimately resulting in uncontrolled inflammation. Thus, reinforcing IBF may be protective and is viewed as therapeutic in this context (Natividad and Verdu, 2012; Duseja and Chawla, 2014).

A significant number of natural compounds have been found to inhibit bacterial growth, although their mechanisms of action frequently remain unclear (Amer et al., 2010). Fructooligosaccharides (FOS) are short-chain oligosaccharides that are generated by hydrolysis of the polysaccharide inulin, which is composed of two to 60 fructose monomers. Inulin is found in different nutrients such as wheat, onion, garlic and banana (Lattimer and Haub, 2010) and is the most common used fiber in prebiotics that, when used in combination with other probiotics, is able to promote the growth of specific beneficial gut bacteria such as bifidobacteria (Gibson et al., 1995; Bosscher et al., 2006).

A number of studies illustrate that FOS and inulin exert a number of different effects on humans and animals. For example, most oraly delivered plant substrate supplements that prevent gastrointestinal infections such as prebiotin^TM^ and symbioram^TM^ contain inulin and FOS, indicating that these compounds are also able to reduce bacterial infection. Moreover, oligosaccharids and in particular inulin and FOS were found to have beneficial effects on intestinal immunity and intestinal barrier function (IBF) (Capitán-Cañadas et al., 2013, 2016). Another study has shown that oligosaccharides from goats milk as well as galactooligosaccharides, modulate cytokine production by intestinal epithelial cells and monocytes *via* a mechanism involving Toll-like receptor 4 (TLR4) (Capitán-Cañadas et al., 2013). TLRs are located on the cell membrane and in endosomes, where they recognize components of cell membranes (TLR2/6, TLR2/TLR4), nucleic acids (TLR3, 7, 8, and 9) and flagellin (TLR5). TLR4 is one of the non-pathogen recognition receptors (PRRs), which are key elements in the communication between the host and the microbiota (Sánchez de Medina et al., 2013). However, further clinical oral applications will require studies on the potential effects of these natural substrates on the human body, which corresponds to the research need addressed in this article.

In addition to the bacterial growth promoting role of inulin and FOS, we reported that FOS inhibited bacterial growth and biofilm formation of *P. aeruginosa* PAO1 (Ortega-González et al., 2014). Additionally, both compounds caused opposing effects on bacterial motility. While FOS inhibited motility, an increased motility was observed in the presence of inulin. Moreover, in co-cultures with eukaryotic cells (macrophages) FOS, and to a lesser extent inulin, reduced the secretion of the inflammatory cytokines IL-6, IL-10, and TNF-α. We were also able to show that the reduction in cytokine secretion is due to a FOS-mediated modulation of the NF-κβ signal transduction pathway (Ortega-González et al., 2014). To gain insight into the detailed molecular processes triggered by FOS and inulin, we report here results from RNA-seq studies.

## Materials and Methods

### Materials

Inulin and FOS were purchased from BENEO-Orafti (Tienen Belgium). Stock solutions at 200 g/L in modified M9 minimal medium were sterilized using 0.22 μm cut-off filters and aliquots were stored at −20°C.

### Culture and Growth Conditions

*Pseudomonas aeruginosa* PAO1 was grown overnight at 37°C in minimum M9 medium. The resulting cultures were then used to inoculate 50 ml of minimum M9 medium supplemented by 5 mM of citrate (MM9) (in 250 ml Erlenmeyer flasks) to an initial OD_600_ of 0.01 and incubated with shaking at 200 rpm at 37°C. When cultures reached OD_600_ = 0.05, FOS or inulin were added to a final concentration of 20 mg/ml and cultures were harvested for analysis 1 h later.

### RNA Extraction, Library Preparation and RNA Sequencing

Total RNA was extracted with the TRI reagent (Ambion) using the manufacturer’s instructions. The RNase inhibitor RiboLock (Fermentas) was added to the samples and DNA was removed by treatment with DNase I (Fermentas). The integrity of the RNA samples was assessed with an Agilent 2100 Bioanalyzer (Agilent Technologies). Subsequently, the 23S, 16S, and 5S rRNAs were removed by subtractive hybridization using the MICROBExpress kit (Ambion) following the protocol reported by Gómez-Lozano et al. (2014). Removal of the rRNA was confirmed by an analysis with an Agilent 2100 Bioanalyzer (Agilent Technologies). Sequencing libraries were prepared using the TruSeq Stranded mRNA Sample Preparation kit (Illumina). After each step, the samples were validated using an Agilent 2100 Bioanalyzer (Agilent Technologies) and the final RNA concentration was measured using a Qubit 2.0 Fluorometer (Invitrogen). The libraries were sequenced using the Illumina HiSeq2000 platform with a paired-end protocol and read lengths of 100 nucleotides.

### RNA-seq Analysis

The quality of sequenced reads was assessed using FastQC software, version 0.11.5 (Andrews and Fast, 2010). Single-end reads were aligned to the reference genome of *P. aeruginosa* PAO1 (GenBank accession number: AE004091.2) using SAMtools v 0.1.19 (Li et al., 2009). BAM files from SAM tools were used as input for the *feature counts* function (Liao et al., 2014) from the Rsubread package (Liao et al., 2013) of Bioconductor version 3.5 to generate a matrix of annotated genes with their corresponding raw counts. An average of 84.5% reads were successfully mapped to the reference genome. The count data were then analyzed to look for differential gene expression levels and statistical significance using DEseq2 (Love et al., 2014; Kimberly and Stephen, 2015).

The threshold to define differences in transcript levels was a statistical Log2 fold change. Genes were considered significantly differentially expressed when *p*-values were below 0.05.

### RNA-Sequencing Data Registration Number

The sequence reads have been deposited in the GEO database under accession N°: GSE124468. The following secure token has been created to allow for the review of record GSE124468: glshocwuzvkprop.

### Analysis of Gene Expression by Quantitative Real Time PCR

Quantitative real time-PCR experiments were performed to validate RNA-seq results. Total RNA was obtained by the TRI reagent^®^ /BCP method (Ambion). One μg of RNA was retrotranscribed following the protocol provided in the manufacturer protocol (iScript BioRad, Alcobendas, Spain) and DNA sequences were amplified with a MX3005P real time PCR instrument (Stratagene) using the primers listed in Supplementary Table S3. The genes of interest were amplified by PCR using the Go Taq@qPCR Master Mix (Promega, Madison, WI, United States) as well as 1 μl of the cDNA template and the primers listed in Supplementary Table S3. Forty PCR cycles were conducted using an annealing temperature of 61°C. The cycle threshold values were normalized to that of the reference transcript, 16S RNA, and data were normalized to the control.

### Generation of Mutants in Genes of the Genomic Island PA0643, PA0644, and PA0646

To generate the PA0643:Gm, PA0644:Gm, and PA0646:Gm mutants 656, 241, and 636 pb DNA fragments, respectively, covering the central part of the genes were amplified by PCR from *P. aeruginosa* PA01 genomic DNA. The resulting products were cloned into plasmid pMBL to yield plasmids pMBL:PA0643, pMBL:PA0644, and pMBL:PA0646. Subsequently, the resulting plasmids were digested with *Bam*HI, which liberated the PA0643, PA0644, and PA0646 fragment. The plasmid pCHESI was also digested with *Bam*HI, to liberate the gentamicin resistance gene (Gm) in order to ligate it with the three DNA fragments. The resulting chimeric DNA was cloned into pMBL digested with *Bam*HI, to yield pMBL:PA0643ΩGm, pMBL:PA0644ΩGm and pMBL:PA0646ΩGm. The resulting plasmids were electroporated into *P. aeruginosa* PA01 for homologous double recombination. Mutant strains were selected on Gm plates and the correctness of the mutation was verified by Southern blotting (Sambrook et al., 1989; Molina-Fuentes et al., 2015).

### Semi-Quantitative Determination of Biofilm Formation

Semi-quantitative determination of biofilm formation were performed as previously described (Christensen et al., 1985). *P. aeruginosa* PA0643, PA0644, and PA0646 mutants were tested in the biofilm-forming capacities in Minimum medium supplemented with 5 mM citrate. The determination of biofilm production was performed after 2, 4, and 6 h of growth by dissolving crystal violet from the biofilm with an ethanol-acetone mixture (70:30) and the absorbance measure at 590 nm.

### Motility Assays

Assays were carried out to determine the effect of the PA0643, PA0644, and PA0646 deletion gene on swimming, twitching and swarming. For swimming assays, bacteria were placed with the help of a sterile tooth-pick at the center of plates containing a 5 mm layer of LB medium with 0.3% (w/v) Bacto agar, 0.2% casamino acids (w/v), and 30 mM glucose. Plates were incubated at 37°C for 24 h and the radial diffusion of bacteria, due to swimming, was inspected. To monitor twitching motility, bacteria were placed with a toothpick into a 2 mm thick layer containing 1.5% (w/v) Bacto agar, 0.2% (w/v) casamino acids, and 30 mM glucose. After incubation at 37°C for 24 h, the expansion of bacteria on the plate was observed. For swarming assays, 5 μl of an overnight culture of bacteria were placed in the center of swarm plates, which are made of 0.5% (w/v) Bacto agar supplemented with 0.2% (w/v) casamino acids and 30 mM glucose. Plates were incubated at 37°C for 24 h, followed by an inspection of the surface movement of the bacteria. All motility assays were performed in triplicate.

### Statistical Analysis

All results are expressed as means from three cultures with the corresponding standard deviations. Data were analyzed for statistical significance using the one-way ANOVA analysis and *a posteriori* least significance test. All analyses were carried out with the SigmaStat 2.03 program (Jandel Corporation, San Rafael, CA, United States). Fitting of dose-response curves was done using Origin 7.0 (OriginLab Corporation, Northampton, MA, United States). Differences were considered significant at *p* < 0.05.

## Results

### FOS and Inulin Induce Differential Changes in *Pseudomonas aeruginosa* Transcript Levels

To understand the cellular response of *P. aeruginosa* PAO1 to FOS and inulin treatment, we conducted RNA-seq studies. Transcriptomic changes were determined in duplicate cultures grown in the absence and in the presence of either FOS or inulin at final concentrations of 20 mg/ml. Between 5,300,000 and up to 7,500,000 reads were obtained for inuline and FOS respectively, of which approximately 85% could be assigned to the 6,322 coding regions of the *P. aeruginosa* PAO1 reference genome (Table 1).

**TABLE 1:** Statistics of RNA-seq data.

**Sample**	**Raw reads**	**Mapped reads**	**Not mapped reads**	**Percent mapped**
MM9-citrate	7472385	6322981	1149404	84.6%
Inulin	5368865	4575351	793514	85.2%
FOS	6598306	5580131	1018175	84.6%

*Shown are means from two replicates.*

**FIGURE 1:**
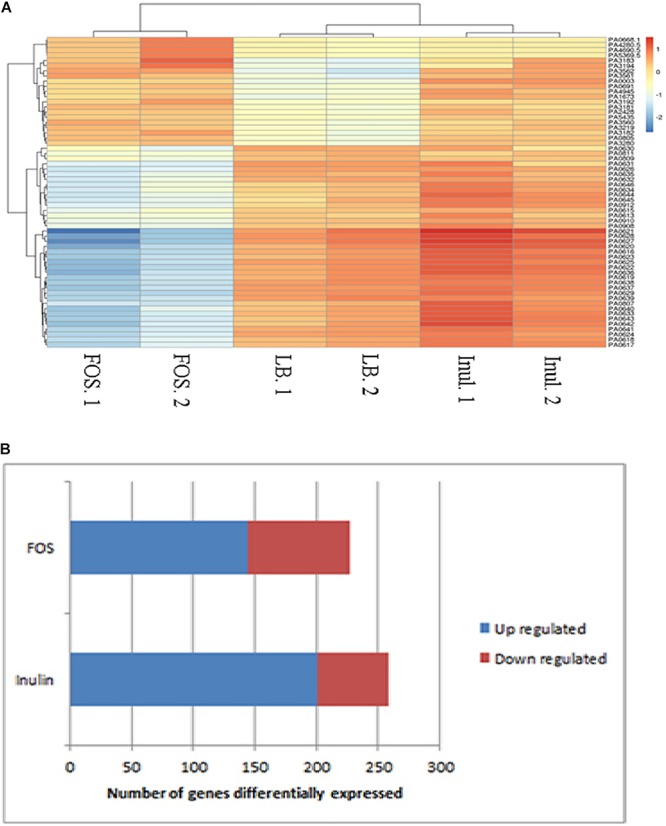
Heat-map **(A)** analysis and hierarchical cluster **(B)** of the genes that are differentially induced and repressed in the presence of FOS or inulin as compared to the untreated control (M9 minimal medium). Blue: genes with increased expression; red: genes with reduced expression.

**FIGURE 2:**
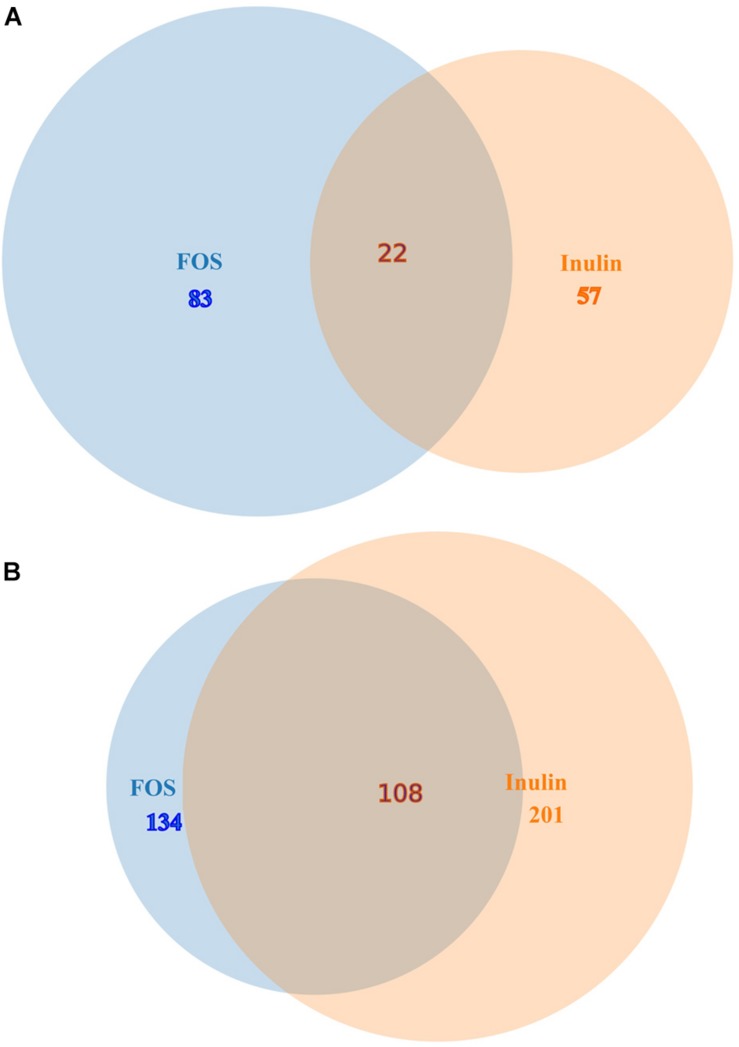
Venn diagrams comparing gene transcript levels in the presence of inulin and FOS. **(A)** Genes down regulated by inulin and FOS. **(B)** Genes up regulated by inulin and FOS.

The heat map shown in Figure 1A illustrates genes with the most important alterations in transcript levels in the presence of FOS/inulin as compared to the control. For both compounds the number of genes with increased transcript levels were superior to those with decreased levels (see Figure 1B). FOS and inulin induced changes in the transcript level of 217 and 258 genes respectively, compared to the control to which no polysaccharide was added (Figure 2). Importantly, down changes in transcript levels of 57 and 83 genes were found to be specific for inulin or FOS, respectively (Figure 2A). Moreover, 201 and 134 genes showed an increase in transcript levels by inulin and FOS, respectively (Figure 2B) indicating that both compounds trigger different changes.

Supplementary Tables S1 and S2 show the list of genes for which the expression level significantly increased or decreased in the presence of 20 mg/ml of inulin (Supplementary Table S1) and FOS (Supplementary Table S2). Analysis of the expression pattern showed that only 22 genes had decreased levels in the presence of both compounds (Figure 2A and Table 2). Among these genes the most prominent changes were observed for genes involved in: (1) Organic acid transport such as PA1342 (*aatj*), which encodes a C4-dicarboxylate transport protein and PA1183 (*dctA*), (2) Central metabolism, like a PA0795 (*prpC*), which regulates a citrate synthase, PA2008 (*fahA*) that controls a fumarylacetoacetase, and PA1585 (*sucA*) encoding a 2- oxoglutatate dehydrogenase, (3) Oxidative stress such as superoxide dismutase PA4366 and (4) Virulence system like the Type VI secretion ATPase (PA0090). These genes were outnumbered by the genes for which both compounds caused an increase in transcript levels (Figure 2 and Table 3).

**TABLE 2:** Transcript levels that were reduced in the presence of both, inulin and FOS.

**Gene Id**	**Gene**	**Protein Function**	**Inulin**		**FOS**	
				
			**log2 fold change**	***p*-value (10^–6^)**	**log2 fold change**	***p*-value (10^–6^)**
PA0090	*clpV1*	Type VI secretion ATPase	−0.9	0.000	−0.8	0.000
PA0296	*spuI*	Glutamylpolyamine synthetase	−0.6	0.008	−0.6	0.007
PA0795	*prpC*	Citrate synthase 2	−1.1	0.001	−0.9	0.002
PA1069	*nd*	Hypothetical protein	−0.7	0.000	−0.5	0.003
PA1171	*sltB2*	Soluble lytic transglycolase	−0.7	0.010	−0.6	0.008
PA1183	*dctA*	C4-dicarboxylate transport protein	−1.5	0.000	−1.2	0.000
PA1342	*aatj*	Probable binding protein component of ABC transporter	−1.0	0.000	−1.1	0.000
PA1585	*sucA*	2-oxoglutarate dehydrogenase	−0.5	0.008	−0.6	0.001
PA1588	*sucC*	Succinyl-CoA synthetase	−0.7	0.000	−0.6	0.002
PA1592	*nd*	Hypothetical protein	−0.9	0.000	−0.8	0.001
PA1787	*acnB*	Aconitate hydratase B	−0.5	0.003	−0.5	0.003
PA2007	*maiA*	Maleylacetatoacetate isomerase	−1.1	0.000	−0.7	0.003
PA2008	*fahA*	Fumarylacetoacetase	−1.1	0.000	−1.1	0.000
PA2040	*pauA4*	Glutamylpolyamine synthetase	−0.9	0.000	−0.7	0.002
PA2247	*bkdA1*	2-oxoisovalerate dehydrogenase	−0.9	0.001	−0.7	0.004
PA4055	*ribC*	Riboflavin synthase	−0.7	0.009	−0.7	0.005
PA4240	*rpsK*	30S ribosomal protein S11	−0.7	0.003	−1.0	0.000
PA4366	*sodB*	Superoxide dismutase	−0.7	0.000	−0.7	0.000
PA4370	*icmP*	Insulin-cleaving metalloproteinase outer membrane protein	−0.8	0.002	−0.9	0.000
PA4578	*nd*	Hypothetical protein	−0.9	0.000	−0.8	0.000
PA4824	*nd*	Hypothetical protein	−0.9	0.000	−0.8	0.000
PA4825	*mgtA*	Mg(2 +) transport ATPase P-type 2	−0.9	0.000	−0.8	0.000

*The above table shows the list of the significant down regulated genes and control with a log2 fold change cut off ≥ 0.5 by taking significant *p-*value < 0.05.*

**TABLE 3:** Transcript levels that were increased in the presence of both, inulin and FOS.

**Gene Id**	**Gene**	**Protein Function**	**Inulin**		**FOS**	
				
			**Log2 fold change**	***p*-value (10^–6^)**	**Log2 fold change**	***p*-value (10^–6^)**
PA0003	*recF*	DNA replication and repair protein	1.8	0.000	1.2	0.000
PA0019	*def*	Peptide deformylase	1.0	0.000	0.8	0.000
PA0026	*plcB*	Phospholipase C	0.7	0.005	0.6	0.007
PA0408	*pilG*	Twitching motility protein	0.8	0.000	0.8	0.000
PA0576	*rpoD*	RNA polymerase sigma factor	0.6	0.000	0.7	0.000
PA0577	*dnaG*	DNA primase	0.6	0.002	0.5	0.003
PA0668	*tyrZ*	Tyrosyl-tRNA synthetase 2	0.9	0.000	0.7	0.000
PA0691	*phdA*	Prevent-host-death protein A	1.6	0.000	1.3	0.000
PA0692	*pdtB*	Phosphate depletion regulated TPS partner B	1.2	0.000	1.1	0.000
PA0693	*exbB2*	Transport protein	1.1	0.000	1.1	0.000
PA0730		3-hydroxyacyl-CoA-acyl carrier protein transferase.	0.8	0.000	0.6	0.004
PA0762	*algU*	RNA polymerase sigma factor	0.6	0.001	0.6	0.000
PA0768	*lepB*	Signal peptidase I	0.7	0.002	0.6	0.004
PA0782	*PutA*	Proline dehydrogenase	0.6	0.002	0.5	0.006
PA0805		Uncharacterized protein	1.2	0.000	1.2	0.000
PA0826		Uncharacterized protein	0.8	0.000	0.7	0.000
PA0842		Probable glycosyl transferase	0.6	0.007	0.6	0.005
PA0896	*aruF*	Arginine *N*-succinyltransferase	0.6	0.001	0.5	0.001
PA0979		Uncharacterized protein	1.2	0.000	1.0	0.000
PA1077	*flgB*	Flagellar basal body rod protein FlgB	0.6	0.007	0.6	0.004
PA1081	*flgF*	Flagellar basal-body rod protein FlgF	0.9	0.000	0.7	0.000
PA1084	*flgl*	Flagellar P-ring protein	0.6	0.000	0.5	0.002
PA1327		Serine protease	0.9	0.000	0.8	0.000
PA1382	*xqhB*	Probable type II secretion system protein	0.6	0.003	0.6	0.004
PA1414		Uncharacterized protein	1.9	0.001	1.7	0.004
PA1606		Uncharacterized protein	0.9	0.000	0.7	0.000
PA1608		Probable chemotaxis transducer	1.1	0.000	0.8	0.000
PA1610	*fabA*	Beta-hydroxydecanoyl-ACP dehydrase	0.8	0.000	0.8	0.000
PA1673		Uncharacterized protein	1.7	0.000	1.3	0.000
PA1796	*folD*	Cyclohydrolase	0.6	0.002	0.7	0.001
PA2022		UDP-glucose 6-dehydrogenase	1.0	0.000	0.9	0.000
PA2428		Uncharacterized protein	1.1	0.000	1.0	0.000
PA2548		Uncharacterized protein	0.8	0.000	0.9	0.000
PA2667	*mvaU*	Biosynthetic process	0.5	0.012	0.6	0.001
PA2738	*himA*	Integration host factor subunit alpha	0.7	0.000	0.8	0.000
PA2882		Probable two-component sensor	0.8	0.001	1.0	0.000
PA2971	*yceD*	Uncharacterized protein	1.0	0.000	0.6	0.003
PA3019	*uup*	Probable ATP-binding component of ABC transporter	0.8	0.000	0.6	0.002
PA3116		Probable aspartate-semialdehyde dehydrogenase	0.6	0.003	0.5	0.008
PA3147	*wbpJ*	Probable glycosyl transferase	0.7	0.001	0.7	0.000
PA3181	*edaA*	2-dehydro-3-deoxy-phosphogluconate aldolase	1.1	0.000	1.4	0.000
PA3183	*zwf*	Glucose-6-phosphate 1-dehydrogenase	1.7	0.000	2.0	0.000
PA3193	*glk*	Glucokinase	0.6	0.004	0.7	0.002
PA3194	*edd*	Phosphogluconate dehydratase	1.5	0.000	1.9	0.000
PA3195	*gapA*	Glyceraldehyde-3-phosphate dehydrogenase	1.0	0.001	1.1	0.000
PA3219		Uncharacterized protein	1.2	0.000	1.3	0.000
PA3280	*oprO*	Pyrophosphate-specific outer membrane porin	1.1	0.000	1.1	0.000
PA3296	*phoA*	Alkaline phosphatase	0.6	0.005	0.6	0.007
PA3305		Uncharacterized protein	2.3	0.001	1.9	0.004
PA3351	*flgM*	Two-component system	0.6	0.009	0.7	0.000
PA3382	*phnE*	Phosphonate transport protein	0.6	0.004	0.7	0.001
PA3383	*phnD*	Binding protein component of ABC phosphonate transporter	0.7	0.000	0.6	0.001
PA3384	*phnC*	Phosphonates import ATP-binding protein	0.9	0.000	0.9	0.000
PA3496	*nd*	Uncharacterized protein	0.5	0.002	0.5	0.007
PA3560	*fruA*	Phosphotransferase system transporter	1.1	0.000	1.3	0.000
PA3561	*fruK*	1-phosphofructokinase	1.8	0.000	1.8	0.000
PA3562	*fruI*	Phosphotransferase system transporter enzyme I	2.0	0.000	2.0	0.000
PA3623	*nd*	Uncharacterized protein	0.7	0.001	0.7	0.004
PA3744	*rimM*	16S rRNA processing protein	0.8	0.000	0.6	0.004
PA3746	*ffh*	Signal recognition particle protein	0.6	0.002	0.5	0.004
PA3903	*prfC*	Peptide chain release factor 3	0.9	0.000	0.7	0.001
PA3990		Uncharacterized protein	1.3	0.000	0.9	0.000
PA4255	*rpmC*	50S ribosomal protein L29	0.9	0.000	0.8	0.000
PA4264	*rpsJ*	30S ribosomal protein S10	0.7	0.001	0.6	0.004
PA4270	*rpoB*	DNA-directed RNA polymerase beta chain	0.4	0.009	0.5	0.003
PA4280	*birA*	Regulation of transcription	0.7	0.000	0.7	0.000
PA4378	*InaA*	Protein of response to stimulus	1.0	0.000	0.6	0.000
PA4418	*ftsI*	Peptidoglycan D.D-transpeptidase	1.1	0.000	0.9	0.000
PA4420	*yabC*	Uncharacterized protein	0.5	0.009	1.0	0.000
PA4421	*yabC*	Uncharacterized protein	1.2	0.000	1.1	0.000
PA4432	*rpsI*	30S ribosomal protein S9	0.7	0.012	0.8	0.000
PA4451	*yrbA*	Uncharacterized protein	0.7	0.000	0.9	0.000
PA4462	*rpoN*	RNA polymerase sigma-54 factor	0.9	0.000	0.6	0.000
PA4520		Probable chemotaxis transducer	0.4	0.009	0.5	0.003
PA4541	*lepA*	Large extracellular protease	0.7	0.000	0.6	0.000
PA4602	*glyA3*	Serine hydroxymethyltransferase	1.0	0.000	0.9	0.000
PA4690		Uncharacterized protein	0.5	0.009	0.6	0.005
PA4723	*dksA*	Suppressor protein	1.0	0.000	0.9	0.000
PA4741	*rpsO*	30S ribosomal protein S15	0.6	0.001	0.9	0.000
PA4747	*secG*	Secretion protein	0.6	0.003	0.7	0.001
PA4844	*ctpL*	Methyl-accepting chemotaxis protein	0.7	0.001	0.6	0.002
PA4853	*fis*	Putative Fis-like DNA-binding protein	0.7	0.006	1.0	0.000
PA4945	*miaA*	Delta 2-isopentenylpyrophosphate	0.8	0.000	0.6	0.004
PA4960	*serB*	Probable phosphoserine phosphatase	0.9	0.000	0.7	0.000
PA4961		Uncharacterized protein	0.7	0.000	0.7	0.000
PA4963		Uncharacterized protein	0.8	0.001	0.7	0.002
PA5013	*ilvE*	Branched-chain-amino-acid transferase	0.7	0.001	0.8	0.000
PA5015	*aceE*	Pyruvate dehydrogenase	0.7	0.001	0.6	0.005
PA5042	*pilO*	Type 4 fimbrial biogenesis protein	1.0	0.000	0.9	0.000
PA5045	*ponA*	Penicillin-binding protein 1A	1.3	0.000	1.0	0.000
PA5058	*phaC2*	Poly(3-hydroxyalkanoic acid) synthase 2	0.8	0.000	1.0	0.000
PA5066	*hisI*	Phosphoribosyl-AMP cyclohydrolase	0.6	0.000	0.5	0.005
PA5152		Probable ATP-binding component of ABC transporter	0.6	0.001	0.6	0.001
PA5170	*arcD*	Arginine/ornithine antiporter	0.7	0.000	0.6	0.000
PA5208		Uncharacterized protein	0.5	0.004	0.4	0.008
PA5235	*glpT*	Glycerol-3-phosphate transporter	1.0	0.000	0.9	0.000
PA5285	*sutA*	Uncharacterized protein	0.6	0.003	0.6	0.002
PA5286	*yjbQ*	Uncharacterized protein	0.7	0.002	0.7	0.003
PA5301	*pauR*	Polyamine catabolic process	0.7	0.002	0.6	0.002
PA5315	*rpmG*	50S ribosomal protein L33	0.6	0.002	0.6	0.001
PA5332	*crc*	Catabolite repression control protein	1.1	0.000	0.8	0.004
PA5348	*nd*	Probable DNA-binding protein	0.6	0.010	0.7	0.005
PA5367	*pstA*	Phosphate ABC transporter	0.9	0.005	1.2	0.000
PA5369	*pstS*	Phosphate ABC transporter	0.8	0.000	0.5	0.006
PA5435	*oadA*	Probable transcarboxylase activity	0.6	0.002	0.9	0.000

*The above table shows the list of the significant up regulated genes and control with a log2 fold change cut off ≥ 0.5 by taking significant *p-*value < 0.05.*

A significant number of these genes appear to be involved in sensing (transcriptional regulators, sensor kinases, and chemotaxis transducers), motility, glucose metabolism as well as control of transcription and protein synthesis (Figure 2 and Table 3).

### Functional Analysis of *Pseudomonas aeruginosa* Transcriptome Following Exposure to FOS and Inulin

**FIGURE 3:**
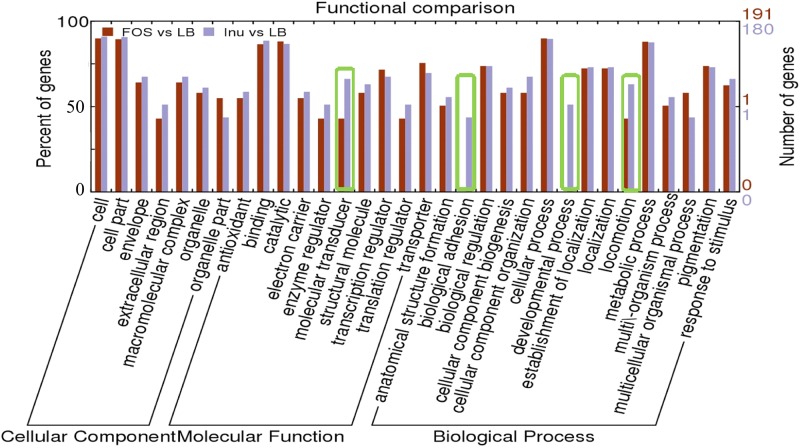
Functional comparison of differentially expressed genes in the presence of FOS and inulin as compared to the control. Functions are organized into three groups: Cellular components, Molecular Function, and Biological Process. The functional groups with the main differences between FOS and inulin are “Molecular transducer,” “Biological adhesion” “Developmental process” and “locomotion system.”

GO terms (WEGO) enrichment analyses were conducted to characterize the DEG (Differentially expressed genes) profiles and K-means clustering was performed to further investigate their biological function. We found that the differentially expressed genes can be classified into 32 categories that belonged to three gene ontology (GO) categories, i.e., the biological process, the cellular component or the molecular function (Figure 3). There were more genes classified into biological processes than the other two categories and most genes were predicted to have a binding function, as these genes are primarily involved in protein metabolism (Figure 3).

The over-expressed DEGs were assigned to 15 GO categories based on biological processes (Figure 3) and the results showed that the response to the stimulus, metabolic process, biological regulation, the establishment of localization and pigmentation were among the most highly represented groups in the biological process category in the presence of inulin or FOS. However, “biological adhesion” and “developmental process” showed a drastic decrease in the number of genes between inulin or FOS samples. While in the “locomotion process” most of the inhibited genes (43%) are annotated in FOS samples (Figure 3).

Furthermore, DEGs were assigned to seven GO categories based on cellular component and the result showed that “Cell” and “Cell part” are similar and highly represented groups for FOS and inulin samples (Figure 3). However, the “extracellular region” and the “organelle part” are lower and distinctly represented in the presence of inulin or FOS (Figure 3).

Genes with altered transcript levels could be grouped into 10 GO terms with different molecular functions of which the categories “antioxidant,” “binding,” “electron carrier,” “transcription regulator,” “structural molecular”, and “transcription regulator” were most populated (Figure 3). It is worth noting that, in the “molecular transducer” category a significantly higher number of genes were noted for inulin as compared to FOS. The individual genes that were classified into these different GO terms are provided in Supplementary Tables S1, S2.

### Differential Gene Expression Pattern in the Presence of FOS

The differential expression analysis performed with DESeq2 in the presence of FOS showed that 83 gene transcript levels were reduced (Table 4), whereas 134 were increased (Figure 2 and Table 5). A large number of genes with reduced transcript levels (43%) were annotated as hypothetical proteins of uncharacterized functions (Table 4). We observed a decrease in the expression of genes related to (1) Metabolic pathways like citrate synthase (*prpC*), glutaminase-asparaginase (*ansB*), glutamylpolyamine synthetase (*pauA4*), and riboflavin synthase (*ribC*), (2) Transport systems such as PA0811 and PA1342 (*aatj*) (3) Translation cellular processes like several ribosomal proteins (*rplQ*, *rpsD*, *rpsK*, and *rplK*) and (4) Virulence such as PA0090 (*clpV*); PA0612 (*ptrB*), PA3866 (*pyocin S4*), and PA4370 (*icmP*) (see Table 4).

**TABLE 4:** Genes with reduced transcript levels following exposure to FOS treatment.

**Gene ID**	**Gene**	**Protein Function**	**log2 Fold Change**	***p*-value (10^–6^)**
PA0049		Uncharacterized protein	−1.2	0.001
PA0090	*clpV1*	Type VI secretion ATPase*	−0.8	0.000
PA0296	*spuI*	Glutamylpolyamine synthetase	−0.6	0.007
PA0612	*ptrB*	Positive regulation of cellular biosynthetic process	−0.9	0.002
PA0613		Uncharacterized protein	−1.3	0.000
PA0614		Uncharacterized protein	−1.1	0.000
PA0615		Uncharacterized protein	−1.3	0.000
PA0616		Uncharacterized protein	−2.5	0.000
PA0617		Uncharacterized protein	−2.1	0.000
PA0618		Uncharacterized protein	−1.9	0.000
PA0619		Uncharacterized protein	−2.4	0.000
PA0620		Uncharacterized protein	−2.5	0.000
PA0621		Uncharacterized protein	−3.2	0.000
PA0622		Uncharacterized protein	−2.5	0.000
PA0623		Uncharacterized protein	−2.3	0.000
PA0624		Uncharacterized protein	−2.3	0.000
PA0625		Uncharacterized protein	−2.5	0.000
PA0626		Uncharacterized protein	−2.1	0.000
PA0627		Uncharacterized protein	−3.7	0.000
PA0628		Uncharacterized protein	−2.9	0.000
PA0629		Uncharacterized protein	−2.6	0.000
PA0630		Uncharacterized protein	−1.8	0.000
PA0631		Uncharacterized protein	−2.9	0.000
PA0632		Uncharacterized protein	−2.1	0.000
PA0633		Uncharacterized protein	−2.2	0.000
PA0634		Uncharacterized protein	−1.5	0.000
PA0635		Uncharacterized protein	−1.9	0.000
PA0636		Uncharacterized protein	−2.5	0.000
PA0637		Uncharacterized protein	−2.8	0.000
PA0638		Uncharacterized protein	−2.6	0.000
PA0639		Uncharacterized protein	−2.5	0.000
PA0640		Uncharacterized protein	−2.1	0.000
PA0641		Uncharacterized protein	−2.1	0.000
PA0642		Uncharacterized protein	−2.2	0.000
PA0643		Uncharacterized protein	−2.2	0.000
PA0644		Uncharacterized protein	−2.0	0.000
PA0645		Uncharacterized protein	−1.9	0.000
PA0646		Uncharacterized protein	−1.5	0.000
PA0647		Uncharacterized protein	−1.3	0.000
PA0795		Uncharacterized protein	−0.9	0.002
PA0807	*ampD*	*N*-acetylmuramoyl-L-alanine amidase	−2.7	0.000
PA0809		Transporter activity	−1.7	0.000
PA0811		Transmembrane transport	−1.8	0.000
PA0812		Uncharacterized protein	−1.7	0.000
PA0908	*alpB*	Response to antibiotic	−2.0	0.000
PA0910	*alpD*	Response to DNA damage stimulus	−1.7	0.000
PA0911	*alpE*	Response to DNA damage stimulus	−1.4	0.000
PA0912		Uncharacterized protein	−2.4	0.000
PA1069		Uncharacterized protein	−0.5	0.003
PA1171	*sltB2*	Soluble lytic transglycolase	−0.6	0.008
PA1183	*dctA*	C4-dicarboxylate transport protein	−1.2	0.000
PA1337	*ansB*	Glutaminase-asparaginase	−0.9	0.002
PA1342	*aatj*	Probable binding protein component of ABC transport	−1.1	0.000
PA1585	*sucA*	2-oxoglutarate dehydrogenase	−0.6	0.001
PA1588	*sucC*	Succinyl-CoA synthetase	−0.6	0.002
PA1592	*nd*	Uncharacterized protein	−0.8	0.001
PA1787	*acnB*	Aconitrate hydratase B	−0.5	0.003
PA2001	*atoB*	Acetyl-CoA acetyltransferase	−1.0	0.002
PA2007	*maiA*	Maleylacetatoacetate isomerase	−0.7	0.003
PA2008	*fahA*	Fumarylacetoacetase	−1.1	0.000
PA2040	*pauA4*	Glutamylpolyamine synthetase	−0.7	0.002
PA2111		Uncharacterized protein	−1.1	0.000
PA2247	*bkdA1*	2-oxoisovalerate dehydrogenase	−0.7	0.004
PA2796	*tal*	Transaldolase	−0.8	0.004
PA3661		Uncharacterized protein	−1.0	0.001
PA3692	*lptF*	Lipotoxon F	−0.8	0.006
PA3866		Pyocin S4	−1.0	0.000
PA4055	*ribC*	Riboflavin synthase	−0.7	0.005
PA4237	*rplQ*	50S ribosomal protein L17	−0.6	0.005
PA4239	*rpsD*	30S ribosomal protein S4	−0.6	0.001
PA4240	*rpsK*	30S ribosomal protein S11	−1.0	0.000
PA4274	*rplk*	50S ribosomal protein L11	−0.7	0.002
PA4366	*sodB*	Superoxide dismutase	−0.7	0.000
PA4370	*icmP*	Insulin-cleaving metalloproteinase outer membrane protein	−0.9	0.000
PA4430		probable cytochrome b	−0.6	0.003
PA4578		Uncharacterized protein	−0.8	0.000
PA4774		Uncharacterized protein	−0.7	0.001
PA4823		Uncharacterized protein	−1.2	0.000
PA4824		Uncharacterized protein	−0.8	0.000
PA4825	*mgtA*	Mg^(2+)^ transport ATPase P-type 2	−0.8	0.000
PA4826		Uncharacterized protein	−0.5	0.005
PA5149	*mviM*	Uncharacterized protein	−1.0	0.005
PA5169	*dctM*	C4-dicarboxylate transport	−1.0	0.001

*The above table shows the list of the significant up regulated genes with a log2 fold change cut off ≥ 0.5 by taking significant *p-*value < 0.05.*

**TABLE 5:** Genes with increased transcript levels following exposure to FOS treatment.

**Gene ID**	**Gene**	**Protein**	**Log2 fold Change**	***p*-value (10^–6^)**
PA0003	*recF*	DNA replication and repair protein	1.2	0.000
PA0019	*def*	Peptide deformylase	0.8	0.000
PA0026	*plcB*	Phospholipase C	0.6	0.007
PA0408	*pilG*	Twitching motility protein	0.8	0.000
PA0576	*rpoD*	RNA polymerase sigma factor	0.7	0.000
PA0577	*dnaG*	DNA primase	0.5	0.003
PA0668	*tyrZ*	Tyrosyl-tRNA synthetase 2	0.7	0.000
PA0691	*phdA*	Prevent-host-death protein A	1.3	0.000
PA0692	*pdtB*	Phosphate depletion regulated TPS partner B	1.1	0.000
PA0693	*exbB2*	Transport protein	1.1	0.000
PA0695		Uncharacterized protein	1.2	0.000
PA0715		Uncharacterized protein	0.6	0.007
PA0730		3-hydroxyacyl-CoA-acyl carrier protein transferase.	0.6	0.004
PA0762	*algU*	RNA polymerase sigma factor	0.6	0.000
PA0768	*lepB*	Signal peptidase I	0.6	0.004
PA0782	*putA*	Proline dehydrogenase	0.5	0.006
PA0805		Uncharacterized protein	1.2	0.000
PA0826		Uncharacterized protein	0.7	0.000
PA0842		Probable glycosyl transferase	0.6	0.005
PA0896	*aruF*	Arginine *N*-succinyltransferase	0.5	0.001
PA0952		Uncharacterized protein	1.1	0.001
PA0979		Uncharacterized protein	1.0	0.000
PA1077	*flgB*	Flagellar basal body rod protein FlgB	0.6	0.004
PA1081	*flgF*	Flagellar basal-body rod protein FlgF	0.7	0.000
PA1084	*flgl*	Flagellar P-ring protein	0.5	0.002
PA1327		Serine protease	0.8	0.000
PA1382	*xqhB*	Probable type II secretion system protein	0.6	0.004
PA1414		Uncharacterized protein	1.7	0.004
PA1606		Uncharacterized protein	0.7	0.000
PA1608		Probable chemotaxis transducer	0.8	0.000
PA1610	*fabA*	Beta-hydroxydecanoyl-ACP dehydrase	0.8	0.000
PA1673		Uncharacterized protein	1.3	0.000
PA1796	*folD*	Cyclohydrolase	0.7	0.001
PA1803	*lon*	Protein secretion by the type III secretion system	0.5	0.003
PA2022		UDP-glucose 6-dehydrogenase	0.9	0.000
PA2426	pvdS	Sigma factor	1.2	0.001
PA2428		Uncharacterized protein	1.0	0.000
PA2461		Uncharacterized protein	0.7	0.008
PA2548		Uncharacterized protein	0.9	0.000
PA2637	nuoA	NADH dehydrogenase I	0.6	0.004
PA2667	*mvaU*	Biosynthetic process	0.6	0.001
PA2685	*vgrG4*	protein secretion system type VI	0.5	0.005
PA2696		probable transcriptional regulator	1.0	0.004
PA2738	*himA*	Integration host factor subunit alpha	0.8	0.000
PA2756		Uncharacterized protein	0.7	0.000
PA2882		Probable two-component sensor	1.0	0.000
PA2971	*yceD*	Uncharacterized protein	0.6	0.003
PA3019	*uup*	Probable ATP-binding component of ABC transporter	0.6	0.002
PA3116		Probableaspartate-semialdehyde dehydrogenase	0.5	0.008
PA3147	*wbpJ*	Probable glycosyl transferase	0.7	0.000
PA3161	*him*	Integration host factor	0.6	0.005
PA3181	*eda*	2-dehydro-3-deoxy-phosphogluconate aldolase	1.4	0.000
PA3182	*pgl*	6-phosphogluconolactonase	2.0	0.000
PA3183	*zwf*	Glucose-6-phosphate 1-dehydrogenase	2.1	0.000
PA3190	*gltB*	Component of ABC sugar transporter	0.8	0.004
PA3192	*gltR*	Two-component response regulator	1.6	0.000
PA3193	*glk*	Glucokinase	0.7	0.002
PA3194	*edd*	Phosphogluconate dehydratase	1.9	0.000
PA3195	*gapA*	Glyceraldehyde-3-phosphate dehydrogenase	1.1	0.000
PA3219		Uncharacterized protein	1.3	0.000
PA3262		Peptidyl-prolyl cis-trans isomerase	0.8	0.001
PA3280	*oprO*	Pyrophosphate-specific outer membrane porin	1.1	0.000
PA3296	*phoA*	Alkaline phosphatase	0.6	0.007
PA3305		Uncharacterized protein	1.9	0.004
PA3345	*hptB*	Histidine phosphotransfer protein	0.5	0.008
PA3351	*flgM*	Two-component system	0.7	0.000
PA3382	*phnE*	Phosphonate transport protein	0.7	0.001
PA3383	*phnD*	Binding protein component of ABC phosphonate transporter	0.6	0.001
PA3384	*phnC*	Phosphonates import ATP-binding protein	0.9	0.000
PA3496		Uncharacterized protein	0.5	0.007
PA3560	*fruA*	Phosphotransferase system transporter	1.3	0.000
PA3561	*fruK*	1-phosphofructokinase	1.8	0.000
PA3562	*fruI*	Phosphotransferase system transporter enzyme I.	2.0	0.000
PA3563		Uncharacterized protein	0.7	0.004
PA3623		Uncharacterized protein	0.6	0.002
PA3744	*rimM*	16S rRNA processing protein	0.6	0.004
PA3746	*ffh*	Signal recognition particle protein	0.5	0.004
PA3903	*prfC*	Peptide chain release factor 3	0.7	0.001
PA3990		Uncharacterized protein	0.9	0.000
PA4255	*rpmC*	50S ribosomal protein L29	1.1	0.000
PA4264	*rpsJ*	30S ribosomal protein S10	0.8	0.000
PA4270	*rpoB*	DNA-directed RNA polymerase beta chain	0.9	0.000
PA4280	*birA*	Regulation of transcription	0.6	0.000
PA4335		Uncharacterized protein	1.1	0.001
PA4336		Uncharacterized protein	0.8	0.003
PA4378	*inaA*	Protein of response to stimulus	0.6	0.004
PA4418	*ftsI*	Peptidoglycan D.D-transpeptidase	0.5	0.003
PA4420	*yabC*	Uncharacterized protein	0.6	0.000
PA4421	*yabC*	Uncharacterized protein	0.9	0.000
PA4432	*rpsI*	30S ribosomal protein S9	0.6	0.005
PA4451	*yrbA*	Uncharacterized protein	0.9	0.000
PA4462	*rpoN*	RNA polymerase sigma-54 factor	0.9	0.000
PA4475		Uncharacterized protein	0.7	0.001
PA4520		Probable chemotaxis transducer	0.6	0.002
PA4525	pilA	Type 4 fimbrial precursor	0.9	0.000
PA4541	*lepA*	Large extracellular protease	1.0	0.000
PA4545	*comL*	Outer membrane lipoprotein	0.6	0.003
PA4602	*glyA3*	Serine hydroxymethyltransferase	0.6	0.004
PA4611		Uncharacterized protein	0.6	0.008
PA4690		Uncharacterized protein	1.1	0.000
PA4723	*dksA*	Suppressor protein	0.7	0.000
PA4741	*rpsO*	30S ribosomal protein S15	0.7	0.002
PA4747	*secG*	Secretion protein	0.8	0.000
PA4844	*ctpL*	Chemoreceptor for inorganic phosphate	0.6	0.005
PA4853	*fis*	Putative Fis-like DNA-binding protein	0.9	0.000
PA4941	*hflC*	Protease	0.5	0.004
PA4944	*hfq*	Motilities and Quorum sensing	0.6	0.001
PA4945	*miaA*	Delta 2-isopentenylpyrophosphate	1.0	0.000
PA4960	*serB*	Probable phosphoserine phosphatase	0.6	0.003
PA4961		Uncharacterized protein	0.5	0.005
PA4963		Uncharacterized protein	0.6	0.001
PA5013	*ilvE*	Branched-chain-amino-acid transferase	0.6	0.000
PA5015	*aceE*	Pyruvate dehydrogenase	0.4	0.008
PA5040	*pilQ*	Type IV pilus-dependent motility	0.5	0.004
PA5042	*pilO*	Type IV pilus-dependent motility	0.9	0.000
PA5043	*pilN*	Type IV pilus-dependent motility	0.7	0.005
PA5045	*ponA*	Penicillin-binding protein 1A	0.6	0.002
PA5058	*phaC2*	Poly(3-hydroxyalkanoic acid) synthase 2	0.7	0.003
PA5066	*hisI*	Phosphoribosyl-AMP cyclohydrolase	0.6	0.002
PA5152		Probable ATP-binding component of ABC transporter	0.6	0.001
PA5170	*arcD*	Arginine/ornithine antiporter	0.8	0.004
PA5208		Uncharacterized protein	0.7	0.005
PA5235	*glpT*	Glycerol-3-phosphate transporter	1.2	0.000
PA5255	algQ	Alginate regulatory protein	0.6	0.007
PA5285	*sutA*	Uncharacterized protein	0.5	0.006
PA5286	*yjbQ*	Uncharacterized protein	0.7	0.000
PA5288	*glnK*	Nitrogen regulatory protein	0.6	0.003
PA5301	*pauR*	Polyamine catabolic process	0.9	0.000
PA5315	*rpmG*	50S ribosomal protein L33	0.7	0.000
PA5332	*crc*	Catabolite repression control protein	0.7	0.000
PA5348		Probable DNA-binding protein	0.7	0.001
PA5367	*pstA*	Phosphate ABC transporter	0.7	0.001
PA5369	*pstS*	Phosphate ABC transporter	0.7	0.000
PA5435	*oadA*	Probable transcarboxylase activity	0.9	0.000

*The above table shows the list of the significant up regulated genes with a log2 fold change cut off ≥ 0.5 by taking significant *p-*value < 0.05.*

Surprisingly, in addition to increasing the expression of genes involved in different metabolic pathways necessary for bacterial growth, we found that transcript levels of many genes that are related to *P. aeruginosa* motility were increased in the presence of FOS (Table 5), exemplified by genes involved in twitching motility (*pilG*), components of the flagellar motor or chemoreceptors including CtpL that mediates specific taxis to inorganic phosphate – a key regulator of *P. aeruginosa* virulence (Zaborin et al., 2009; Bains et al., 2012). In the same manner, transcript levels of many genes associated within the glucose metabolism were also increased including genes *eda*, *zwf*, *gapA, glk, edd*, or *gltR* (Table 5) suggesting that *P. aeruginosa* can catabolize these compounds.

### Effect of FOS on Virulence Related Gene Transcript Levels

All the DEGs were mapped to KO terms in the KEGG database to identify FOS modulated genes that play a role in bacterial virulence, motility, or sensitivity to antibiotics and the corresponding genes are provided in Table 6.

**TABLE 6:** Genes with increased transcript levels in response to FOS that are related to bacterial pathogenicity.

**Gene ID**	**Gene**	**Protein**	**Log2 Fold Change**	**Function**	**References**
PA0408	*pilG*	Twitching motility protein	0.81	Twitching motility and Chemotaxis	Darzins, 1993; Darzins and Russell, 1997; Whitchurch et al., 2004
PA0668	*tyrZ*	Tyrosyl-tRNA synthetase 2	0.7	Inhibit growth and biofilm formation	Williams-Wagner et al., 2015
PA0692	*pdtB*	Phosphate depletion regulated TPS partner B	1.1	Type V secretion system	Faure et al., 2014
PA0762	*algU*	RNA polymerase sigma factor	0.6	Control expression of virulence genes	Stacey and Pritchett, 2016; Stacey et al., 2017
PA1077	*flgB*	Flagellar basal-body rod protein	0.63	Bacterial-type flagellum-dependent cell motility	Goodier and Ahmer, 2001
PA1081	*flgF*	Flagellar basal-body rod protein FlgF	0.75	Flagellar assembly	Goodier and Ahmer, 2001
PA1084	*flgI*	Flagellar P-ring protein	0.54	Flagellar assembly	Goodier and Ahmer, 2001
PA1382	*xqhB*	Probable type II secretion system protein	0.6	Type II secretion system	Stover et al., 2000
PA1608		Probable chemotaxis transducer	0.8	Chemotaxis system and biofilm formation	Southey-Pillig et al., 2005
PA2426	*pvdS*	Sigma factor	1.22	Sigma factor activity	Tiburzi et al., 2008
PA3183	*zwf*	Glucose-6-phosphate 1-dehydrogenase	2.13	Carbohydrate metabolic pathway	Udaondo et al., 2018
PA3192	*gltR*	Two component system	1.6	Carbohydrate metabolic and virulence	Daddaoua et al., 2014
PA3351	*flgM*	Negative regulator of flagellin synthesis FlgM	0.73	Flagellar assembly	Goodier and Ahmer, 2001
PA4520		Chemotaxis transducer	0.6	Chemotaxis system	Victoria et al., 2003
PA4747	*secG*	Secretion protein	0.7	Sec secretory pathway	Crowther et al., 2015
PA4844	*ctpL*	Chemoreceptor for inorganic phosphate	0.6	Chemotaxis to inorganic phosphate	Rico-Jiménez et al., 2016
PA5040	*pilQ*	Type 4 fimbrial biogenesis protein	0.5	Surface filaments involved in host colonization	Darzins, 1993; Darzins and Russell, 1997; Whitchurch et al., 2004
PA5042	*pilO*	Type 4 fimbrial biogenesis protein	0.9	Surface filaments involved in adhesion cell, twitching motility	Martin et al., 1995; Leighton et al., 2018; Ayers et al., 2009
PA5043	*pilN*	Type 4 fimbrial biogenesis protein	0.7	Surface filaments involved in host colonization	Ayers et al., 2009
PA5045	*ponA*	Penicillin-binding protein 1A	1.0	*ampR*-β-lactamase modulation	Handfield et al., 1997
PA5332	*crc*	Catabolite repression control protein	0.8	Virulence, Quorum Sensing	Milojevic et al., 2013; Chakravarthy et al., 2017
PA5435	*oadA*	Probable transcarboxylase activity	0.9	Virulence	Bains et al., 2012
PA5369	*pstS*	Phosphate ABC transporter transporter control protein	0.8	Virulence	Zaborin et al., 2009

Noteworthy are the increases observed for transcript levels of PA0668 which encodes a TyrZ: Tyrosyl-tRNA synthetase2 that inhibits growth and biofilm formation (Williams-Wagner et al., 2015), genes of the *pilMNOPQ* operon, which are important for the pilus assembly system (T4P) and promote surface-associated attachment (Ayers et al., 2009; Tammam et al., 2013; McCallum et al., 2016) showed increased transcript levels like *pilG* (PA0408) (Log2 fold = 0.8); *pilQ* (PA5040) (0.5 Log2 fold); *pilO* (PA5042) (0.9 Log2 fold); *pilN* (PA5043) (0.7 Log2 fold); or different genes of the *flg* operon that encode proteins of the flagellar motor such as *flgB* (PA1077) (0.6 Log2 fold); *flgF* (PA1081) (0.7 Log2 fold); *flgI* (PA1084) (0.5 Log2 fold); and *flgM* (PA3351) (0.7 Log2 fold). Moreover, the transcript levels of three chemoreceptors were increased in the presence of FOS, of which two (PA1608, PA4520) are of unknown function, whereas PA4844 (*ctpl*) encodes a specific chemoreceptor for inorganic phosphate (Rico-Jiménez et al., 2016). In addition, the *zwf1* gene encoding a glucose-6-phosphate dehydrogenase that is involved in *P. aeruginosa* virulence (Udaondo et al., 2018) showed much higher transcript levels (2.13 Log2 fold) in the presence of FOS. In the same manner, transcript levels of *gltR* encoding the response regulator of the GtrS/GltR two component system (TCS) were significantly increased (1.6 Log2 fold) (Table 6). This TCS was found to regulate the expression of *toxA* encoding the primary virulence factor exotoxin A (Udaondo et al., 2018).

However, the results (Table 7) show several virulence related genes with reduced transcript levels, such as PA0090 encoding the ClpV1 protein, involved in the type VI secretion system (Bönemann et al., 2009), or the PtrB component of the type III secretion system (TTSS) that coordinates TTSS repression and pyocin synthesis under DNA damage (Weihui and Shouguang, 2005). The latter observation agrees with the reduced transcript level of the gene (PA3866) encoding the pyocin S4 (Ameer et al., 2012).

**TABLE 7:** Genes with decreased transcript levels in response to FOS that are related to bacterial pathogenicity.

**Gene ID**	**Gene**	**Protein**	**Fold change**	**Function**	**References**
PA0090	*clpV*	Type VI secretion ATPase	−0.84	Biofilm formation and secretion system type VI	Bönemann et al., 2009
PA0296	*spuI*	Glutamylpolyamine synthetase	−0.55	Polyamine toxicity	Dong-Kwon and Chung-Dar, 2006
PA0612	*ptrB*	Protease	−0.85	Suppresses the Type III Secretion System	Weihui and Shouguang, 2005
PA0807	*ampD*	*N*-acetylmuramoyl-L-alanine amidase	−2.68	β-lactam resistance	Amber and Nancy Hanson, 2008
PA0908	*alpB*	Outer membrane protein AlpB	−1.98	Cellular response to antibiotic	Alvarez-Ortega et al., 2010
PA1183	*dctA*	C4-dicarboxylate transport protein	−1.23	Growth process	Valentini et al., 2011
PA3692	*lptF*	Lipotoxon F	−0.78	Integral component of membrane and survival factor	Heath-Damron et al., 2009
PA3866		*Pyocin S4*, soluble (S-type) pyocins	−0.95	Virulence factor	Ameer et al., 2012
PA4370	*icmP*	Insulin-cleaving metalloproteinase outer membrane protein precursor	−0.93	Pathogenicity	Christian et al., 2015

In addition, the results shown in Table 7 reveal reduced transcript levels for PA3692 (*lptF*), encoding an outer membrane protein (alipotoxon), which plays a key role in *P. aeruginosa* survival under harsh environmental conditions, including lung colonization in patients with cystic fibrosis (Heath-Damron et al., 2009). Furthermore, the reduction in transcript levels for PA0807 (*ampD*) and PA0908 (*alpB*), encoding proteins involved in responses to antibiotics, or in PA0296, involved in polyamine toxicity, may indicate that FOS modulates sensitivity to antibiotics (Amber and Nancy Hanson, 2008; Shah and Swiatlo, 2008; Alvarez-Ortega et al., 2010) and polyamines (Dong-Kwon and Chung-Dar, 2006). These data have been confirmed by real time quantitative PCR (rt-qPCR) experiments (Table 8).

**TABLE 8:** Quantitative real time PCR experiments to quantify the effect of FOS on the transcript levels of genes that belong to the genomic island and that are related to bacterial pathogenicity in *P. aeruginosa*.

**Gene ID**	**Gene**	**Protein**	**Relative expression levels**	***P*-value**
PA0807	*ampD*	*N*-acetylmuramoyl-L-alanine amidase	−6.1	0.04
PA0908	*alpB*	Outer membrane protein AlpB	−5.8	0.04
PA1183	*dctA*	C4-dicarboxylate transport protein	−7.2	0.02
PA0296	*spuI*	Glutamylpolyamine synthetase	−7.1	0.04
PA3866	*Pyocin S4*	Soluble (S-type) pyocins	−10.7	0.01
PA4370	*icmP*	Insulin-cleaving metalloproteinase outer membrane	−10.4	0.02
PA4844	*ctpL*	Chemoreceptor for inorganic phosphate	+ 0.65	0.09

*Experiments were conducted using *P. aeruginosa* cultures grown in the presence and absence of 20 mg/ml of FOS. The cycle threshold values were normalized to that of the reference transcript, 16S RNA, and data of relative expression levels were normalized to the control lacking FOS.*

Moreover, FOS treatment resulted in lower transcript levels of the *dctA* gene (PA1183) which is associated with the normal growth of *P. aeruginosa* (Valentini et al., 2011) and of the *icmP* gene (PA4370) encoding an metalloproteinase outer membrane protein, which has been shown to degrade the plasminogen activator (Christian et al., 2015) and plays a key role in the *Pseudomonas aeruginosa* pathogenicity. These FOS mediated alterations in transcript levels have been confirmed by rt-qPCR studies (see Table 8). Altogether, data suggest that FOS acts as a signal molecule that modulates bacterial virulence through distinct signaling pathways.

### Confirmation That FOS Reduces Expression of Structural Proteins of Secretion System III and VI

To confirm that FOS mediates changes in secretion system genes, we have conducted rt-qPCR experiments to determine the influence of FOS and inulin on the expression of 4 genes that encode proteins that are part of type III and VI secretion systems. The products of the *pcrV* and *exsA* genes control the activation of the type III secretion system (Lee et al., 2010), whereas the proteins encoded by *hcp1* and *vgrG1* are necessary for the type VI secretion system (Hachani et al., 2011).

**FIGURE 4:**
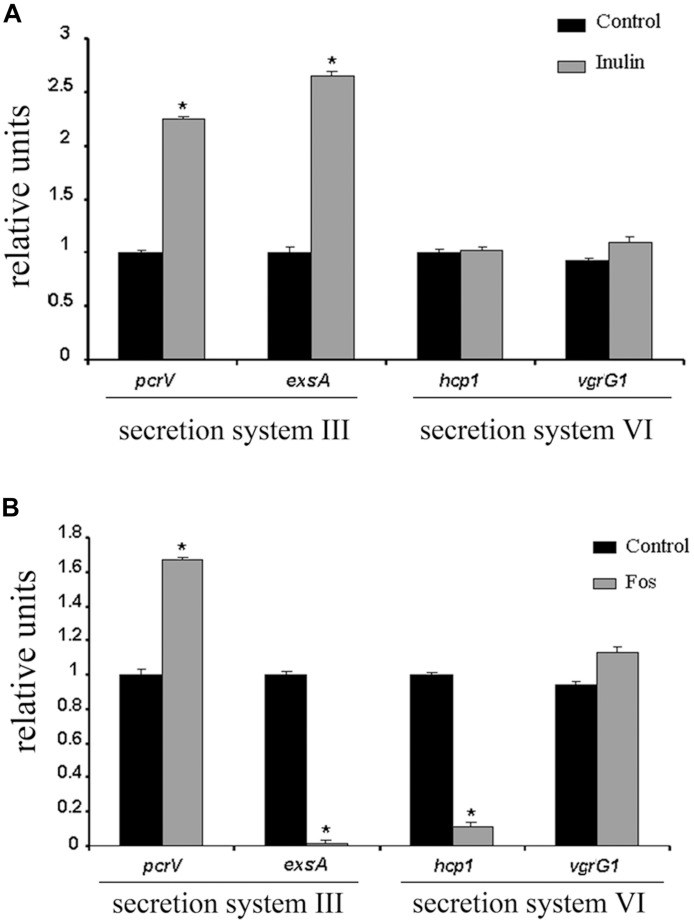
Quantitative RT-PCR validations of the effect of FOS and inulin on the expression of secretion system genes. rt-qPCR studies of *P. aeruginosa* of cultures grown in the presence and absence of 20 mg/ml inulin **(A)** or FOS **(B)**. The expression of genes encoding proteins of secretion systems III and VI are shown. *P < 0.05 vs. Control.

We found that inulin caused a significant increase in *pcrV* and *exsA* transcript levels, whereas those of *hcp1* and *vgrG1* did not vary (Figure 4A). In contrast, the expression of *exsA* and *hcp1* were dramatically down regulated by factors of approximately 20 and 7, respectively, in the presence of FOS (Figure 4B). FOS but not inulin down regulated the expression of two components of the type III and VI secretion system.

### Identification of Genomics Islands Widely Repressed by FOS-Treatment in *Pseudomonas aeruginosa* PAO1

Due to their relevance to human health, extensive efforts have been made to study genomic islands, which are large genetic elements acquired through horizontal transmission (Juhas et al., 2009; Stephen and Julian, 2015; Mao and Lu, 2016).

**FIGURE 5:**
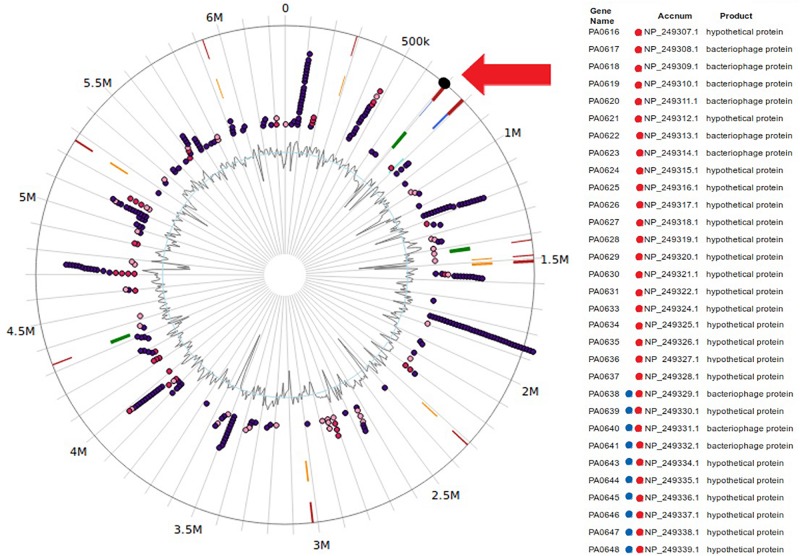
Location of the genomics island with genes widely repressed by FOS-treatment in *Pseudomonas aeruginosa* PAO1 genome. Figure were made using IslandViewer4 (Bertelli et al., 2017). Genes ids with a red dot represent genes repressed in presence of FOS treatment. Genes with blue dot represent genes identified in a genomic island in *P. aeruginosa* PAO1 genome.

**FIGURE 6:**
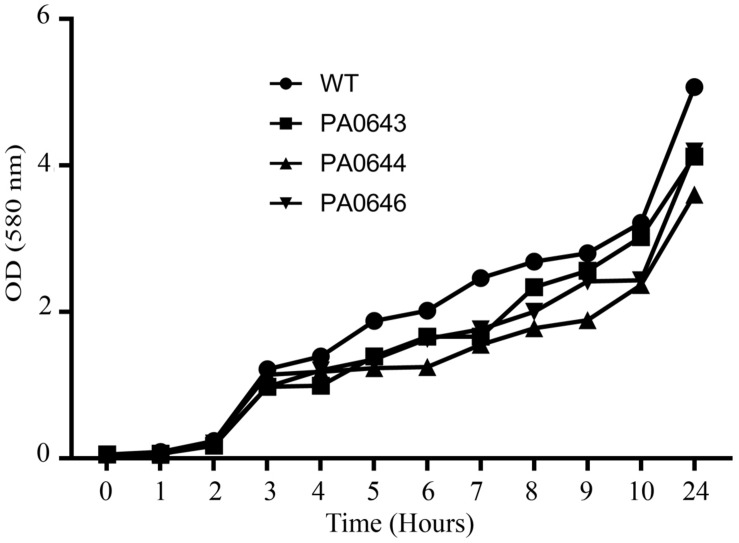
The effect of PA0643, PA0644, and PA0646 deletion on the growth of *P. aeruginosa* PAO1. Shown are results from growth experiments that were conducted in M9 minimum medium at 37°C for 24 h. Representative data from one of three independent experiments with similar results are shown.

**FIGURE 7:**
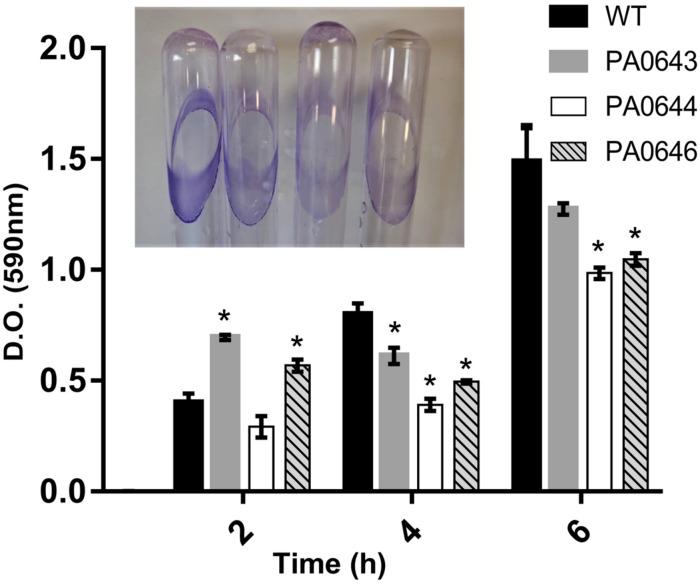
The effect of PA0643, PA0644, and PA0646 deletion on biofilm formation of *P. aeruginosa* PAO1. Biofilm formation was monitored in minimum medium supplemented with 5 mM of citrate and quantified after 2, 4, and 6 h. The OD at 590 nm of crystal violet (CV) stained and resuspended bacteria from biofilms was recorded. Shown are means and standard deviations with *n* = 3–6; *P < 0.05 vs. WT.

**FIGURE 8:**
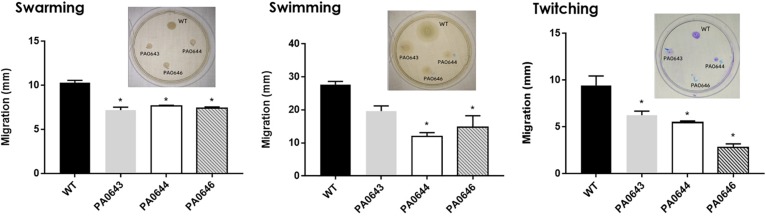
Effect of PA0643, PA0644, and PA0646 deletion on the motility of *P. aeruginosa* PAO1. Motility assays were carried out as described in section “Materials and Methods.” Shown are means and standard deviations with *n* = 3–6; *P < 0.05 vs. WT.

Our data, using RNAseq sequence analysis, indicates the presence of a genomic island in PAO1 of ∼17 kb comprising 15 genes that had been inserted into the 3′-end of the PA0639 gene (Figure 5) through a phage encoded R2/F2 pyocin (Chang et al., 2005). Most of its genes were clearly repressed in the presence of FOS. Unfortunately, most of these genes code for proteins with unknown functions. Furthermore, in order to determine the biological role of this genomic island, various isogenic mutants were constructed and submitted to a phenotypic analysis that investigates changes in growth, biofilm formation, and motility. Interestingly, PA0643, PA0644, and PA0646 mutants showed reduced growth inhibition compared to the wild-type strain (Figure 6). In addition, while the PA0643 mutant strain did not cause any significant changes, the PA0644 and PA0646 isogenic mutant demonstrated a reduction in biofilm formation at 4 and 6 h (Figure 7). Moreover, PA0643, PA0644, and PA0646 mutants were tested for their ability to swim, swarm, and twitch and the results showed that the mutants exhibited differences in all three types of motility (Figure 8). While PA0643, PA0644, and PA0646 mutants caused the same reduced change in swarming motility, it was found that PA0644 and PA0646 mutants significantly inhibited (at least 50–60% of WT) the swimming motility. Interestingly, the twitching motility has been drastically reduced in the case of the PA0646 mutant (Figure 8). Markedly, this is the first study which shows that the deletion of PA0643, PA0644, and PA0646 genes are able to block *P. aeruginosa* swarming, swimming, and twitching motility. However, further studies are required to elucidate the function of these proteins.

## Discussion

Various factors appear to be implicated in the ability of *P. aeruginosa* to cause health problems. While surface structures, including pili and the polysaccharide, seem to favor biofilm formation and adhesion of *P. aeruginosa* to host cells (Pollack, 1984/1992; Prince, 1992) promoting colonization, its exotoxins in combination with various secretion systems deteriorate the host’s defenses. The identification of signal molecules and the study of their corresponding molecular mechanism, which is associated with these processes, are of utmost interest.

Prebiotics have been shown to exert beneficial effects on human health by altering the intestinal microbiota and also by inhibiting the progression of some pathogenic strains (Knol et al., 2005), due to an indirect effect caused by the selective growth of host friendly bacteria. To our knowledge, antimicrobial properties have been described for a number of oligosaccharides (Daddaoua et al., 2006) and in a previous study, it was shown that FOS as a prebiotic had specific effects on *P. aeruginosa*, since it reduced growth, limited the formation of biofilm, impaired motility, and reduced the inflammatory response (Ortega-González et al., 2014). Although, inulin and FOS are structurally related oligosaccharides, they differ in chain length and both compounds can be used as a carbon and energy source for *P*. *aeruginosa* growth.

The present study demonstrates that FOS induces a number of important changes in *P. aeruginosa* transcript levels some of which are related to bacterial survival (Figure 1) and provides an initial insight into the corresponding molecular mechanisms. As shown in Figure 2, in the 162 genes with reduced transcript levels by FOS and inulin, only 13% of them were affected by both compounds. Similarly, of the 443 genes with increased levels only 24% of gene transcript levels were altered by both compounds. Therefore, these compounds can be considered as signal molecules; however, their molecular mechanisms remain unknown.

RNA-Seq and rt-qPCR based comparative RNA profiling of *P. aeruginosa* PAO1 in the presence of FOS or inulin, not only highlighted known functions required for survival metabolism, but revealed a decrease in transcript levels of genes associated with carbohydrate metabolism and growth such as PA1183 (*dctA)* which has been shown to be associated previously with bacterial growth (Valentini et al., 2011). Thus, the growth reduction may be due to a reduction in transcript levels of genes associated with carboxylic acid transport, carboxylic acid metabolism, and reduction in ribosomal proteins.

Furthermore, these studies revealed a decrease of *spuI* gene transcript levels (PA0296), confirmed by rt-qPCR (Table 8), which control the expression of polyamine toxicity and of pyocins (PA3866), which are virulence factors that are induced by DNA-damaging agents, such as UV light and mitomycin C (Dean-Scholl and Martin, 2008).

The current analysis clearly shows that the pathogenesis of *P*. *aeruginosa* in the presence of FOS is compromised due to a decrease in several virulence-associated genes; i.e., *alpB* (PA0908), a holin-like protein that is required for lysis and the *icmP* gene (PA4370), which has been shown to degrade plasminogen activator (Christian et al., 2015) and may play a role in *P. aeruginosa* pathogenicity (Tables 5, 8).

Secretion of exotoxins through type III secretion systems (T3SSs) (Filloux, 2011) are related to acute infections in *P. aeruginosa*, while type VI secretion systems are often associated with chronic infections and biofilm formation (Silverman et al., 2012).

Here we show by rt-qPCR that FOS lowers the transcript levels of *exsA* encoding a transcriptional activator of the secretion system type (T3SS) as well as those of a key protein necessary for the secretion system type VI (T6SS) function, encoded by the *hcp* gene (Figure 4).

Interestingly, the RNA seq analysis data coincide with a report that demonstrated that the repression of the *ptrB* gene (PA0612) is implicated in the regulation of the T3SS under DNA damage stress conditions (Weihui and Shouguang, 2005). In addition, this report demonstrates that a repression of *clpV* (PA0090) regulates the biofilm formation and T6SS (Table 5). Altogether, our study is consistent with the notion that the FOS mediated reduction in pathogenicity is mediated by the secretion systems III and VI.

Further, we obtained initial data suggesting that FOS modulates bacterial resistance to antibiotics, since FOS reduced the expression of *ampD* (PA0807) transcription levels which leads to the constitutive hyperproduction of the beta-lactamase AmpC and consequently to an increase of the β-lactam resistance (Lindberg et al., 1987). Furthermore, the data shows that FOS lowers transcript levels of *alpB* (PA0908) a gene product which is related to cell responses to antibiotics (Alvarez-Ortega et al., 2010).

The RNA seq analysis demonstrated the presence of an island with different genes of unknown function, whose expression was completely inhibited in the presence of FOS (Figure 5 and Table 4). The deletion of the PA0643, PA0644, and PA0646 genes caused alterations in bacterial growth (Figure 6), biofilm formation (Figure 7) and motility (Figure 8). The alteration of the transcript levels of this island is thus another possible mechanism by which FOS reduces bacterial pathogenesis.

## Conclusion

FOS containing supplements are currently being used to prevent gastrointestinal infections (Yasuda et al., 2012), suggesting that it is a valid strategy to combat *Pseudomonas* infection by potentially including FOS in antimicrobial cocktails. The present study is a contribution to close the gap of knowledge that exists in the corresponding molecular mechanisms.

## Data Availability Statement

All datasets generated for this study are included in the article/Supplementary Material.

## Author Contributions

JR-G, CS, and MG provided advice on experimental design. ZU and CS collected and assembled the data. AD designed the study, supervised and analyzed the data, and wrote the manuscript. TK and J-LR revised the manuscript. All authors reviewed and commented on the manuscript.

## Conflict of Interest

The authors declare that the research was conducted in the absence of any commercial or financial relationships that could be construed as a potential conflict of interest.
