# Supplementary material for: Full Transcriptomic Response of Pseudomonas aeruginosa to an Inulin-Derived Fructooligosaccharide
Source: Front Microbiol. 2020 Feb 20;11:202. doi: 10.3389/fmicb.2020.00202 (PMC7044273; doi:10.3389/fmicb.2020.00202)
Supplement: Supplementary file 3 [file Table_3.pdf]

Supl. Table S3. Sequences of primers used in this study.

Primer	Sequence	Purpose
**pcrV.1f**	5' CAGTGCCGAGCAGGAACTG-3’	Quantification of transcript levels by RT-qPCR
**pcrV.1r**	5'-CTGAAATAGGCCGACACCAGGAAC-3’	Gene which regulated system secretion type III
**exsA.1f**	5'-AGGGTAAACAAGGAAGAGGGCGTATAT-3’	Gene which regulated system secretion type III
**exAs.1r**	5'-CTGGACGAAGCCTTGTAGAAACTGG-3’	Gene which regulated system secretion type III
**hcp.1f**	5'-GTCAAGGGTGAGTCCAAGGACAAGAC-3’	Gene which regulated system secretion type VI
**hcp.1r**	5'-CATCAGGTTGGGCGTGGACTTG-3’	Gene which regulated system secretion type VI
**vrgG.1f**	5'-AGAACCAGAGCGTGCCGGAGA-3’	Gene which regulated system secretion type VI
**vrgG.1r**	5'-CCAGTAGTAGATGC CTTCCTGTTCCAT-3’	Gene which regulated system secretion type VI
**16S. f**	5'-ATTACCGCGGCTGCTGGCA-3’	Normalization of gene expression
**16S. R**	5'-CCTACGGGAGGCAGCAG-3’	Normalization of gene expression
**PA0643mut.f**	5'-ATGGCTTGGCATTCGAAAGGTTCGGTTTCC-3′	Generation of PA0643 mutant
**PA0643mut.r**	5'-TCAATTCCACCTCCCCATGGCGACATAGC-3′	Generation of PA0643 mutant
**PA0644mut.f**	5'-ATGAAGCTGTTGTTGAAACCTTTGTTGCAGCAGG-3′	Generation of PA0644 mutant
**PA0644mut.r**	5'-TTATCCCATTGGATGTTCATAGCTGAACGCTACCTC-3′	Generation of PA0644 mutant
**PA0646mut.f**	5'-ATGCCTTGGTATTCCACAGGCACGGTTTCC-3′	Generation of PA0646 mutant
**PA0646mut.r**	5'-TCAGTACCACCTCCCCACAGCCATCACCG-3′	Generation of PA0646 mutant
**qPCRPA0807.f**	5'-CAGCGCATCTTCAACCTGGTCGCCGAG-3′	Quantification of transcript levels by RT-qPCR
**qPCRPA0807.r**	5'-GTCATGTCCGGGTAGCGCTGCAGGATGTTCTT-3′	Quantification of transcript levels by RT-qPCR
**qPCRPA0612.f**	5'-CTGACCTTGCCGATCACGCCAACGAACTG-3′	Quantification of transcript levels by RT-qPCR
**qPCRPA0612.r**	5'-GGTCCTGGCAGTCGATGCAGCGACTGC-3′	Quantification of transcript levels by RT-qPCR
**qPCRPA0908.f**	5'-ACCTGCCGGGTATCGATGGCAACGC-3′	Quantification of transcript levels by RT-qPCR
**qPCRPA0908.r**	5'-GCAGAGGGTCGCGGCGACGAAGG-3′	Quantification of transcript levels by RT-qPCR
**qPCRPA1183.f**	5'-AAGATCGCTGCCTACGCCGCTGCCG-3′	Quantification of transcript levels by RT-qPCR
**qPCRPA1183.r**	5'-CATCACGTGGGAGACGCGCTCGATGAAC-3′	Quantification of transcript levels by RT-qPCR
**qPCRPA0296.f**	5'-GCTGCTCAAGCGCCTGGTCAAGAACATCG-3′	Quantification of transcript levels by RT-qPCR
**qPCRPA0296.r**	5'-GGCAGGGTCTCGAGCACACCGCCG-3′	Quantification of transcript levels by RT-qPCR
**qPCRPA3866.f**	5'-CGGAATCAGATTGCCTTGGCAGCACAGG-3′	Quantification of transcript levels by RT-qPCR
**qPCRPA3866.r**	5'-CCTTGACCTGGGCGGTTAGCCTGTTGACC-3′	Quantification of transcript levels by RT-qPCR
**qPCRPA4370.f**	5'-TTAACGCCTGGCCGCTGGACGAAGGC-3′	Quantification of transcript levels by RT-qPCR
**qPCRPA4370.r**	5'-CCCCAGAGGAGGAACTCGATGGCGTGGT-3′	Quantification of transcript levels by RT-qPCR
**qPCRPA4844.f**	5'- CGCCTGATCCAGGGTGGGATGATCGC -3′	Quantification of transcript levels by RT-qPCR
**qPCRPA4844.r**	5'- CGAGGAAGCTGCGCAGGCGGTTCAG -3′	Quantification of transcript levels

		by RT-qPCR
